# Monoamine Oxidase B (MAO-B) as an Inducer of Mitochondrial Reactive Oxygen Species (ROS) Production and Myofibroblast Differentiation in Cardiac Fibroblasts of Mice

**DOI:** 10.3390/cells15100881

**Published:** 2026-05-12

**Authors:** Gerhild Euler, Hannah Disch, Maximilian Trautmann, Anne Bernhardt, Jennifer Krechmeier, Rainer Schulz, Jacqueline Heger

**Affiliations:** Institute of Physiology, Justus Liebig University, 35392 Giessen, Germany; hannah.n.disch@med.uni-giessen.de (H.D.); maximilian-andreas.trautmann@med.uni-giessen.de (M.T.); anne.bernhardt@gmx.de (A.B.); jennifer.krechmeier@med.uni-giessen.de (J.K.); rainer.schulz@physiologie.med.uni-giessen.de (R.S.); jacqueline.heger@physiologie.med.uni-giessen.de (J.H.)

**Keywords:** fibroblasts, fibrosis, heart, mitochondria, reactive oxygen species

## Abstract

**Highlights:**

**What are the main findings?**

- Monoamine oxidase B (MAO-B) accelerates mitochondrial ROS production in cardiac fibroblasts.
- MAO-B provokes myofibroblast differentiation.

**What are the implications of the main findings?**

- The induction of MAO-B in ischemia/reperfusion or under pressure overload may contribute to cardiac fibrosis.
- Specific pharmacologic inhibition of MAO-B can be an option in the treatment of detrimental cardiac fibrosis.

**Abstract:**

MAO-B-specific inhibition, either in knockout (KO) mice or pharmacologically, preserves left ventricular function and reduces cardiac fibrosis after myocardial infarction or pressure overload. We investigated whether stimulation of MAO-B in cardiac fibroblasts provokes ROS production and myofibroblast development. Fibroblast-specific MAO-B knockdown (KD) mice were created by crossing Col1a2CreERT mice with MAO-B^fl/fl^ mice. The KD was induced by tamoxifen injection. Fibroblasts of KD mice and wild types (WTs) were isolated and reduced MAO-B expression in KD fibroblasts was confirmed. In isolated mitochondria from the left ventricle of these mice, ROS production was reduced under stimulation with the specific MAO-B substrate β-phenylethylamine (PEA). Mitochondrial ROS production in fibroblasts, detected by MitoSox Red staining, increased under PEA (1000 µM) stimulation only in WT fibroblasts. mRNA of the marker genes for myofibroblast differentiation, Col1a1 and periostin, increased 2- or 3-fold, respectively, in WT but not in MAO-B KD fibroblasts. The enhanced migration potential under PEA was reduced in MAO-B KD fibroblasts. In conclusion, stimulation of MAO-B in cardiac fibroblasts leads to the formation of mitochondrial ROS, enhancement of myofibroblast marker gene expression and migration of the cells. Excessive fibrosis caused by elevated MAO-B activity in myocardial infarction can therefore contribute to cardiac dysfunction.

## 1. Introduction

Cardiac disease, independent of its primary origin, like inflammation, hypertension, or ischemia/reperfusion, is one of the most concerning illnesses worldwide, often leading to heart failure, which affects approximately 70 million people globally. Despite advances in the management of cardiac diseases, heart failure remains a leading cause of cardiovascular morbidity and mortality [1]. A better understanding of the triggers of the disease and therapeutic improvements are therefore still urgently needed.

Reactive oxygen species (ROS) are one of the central molecules involved in the onset and progression of cardiac diseases. In cardiomyocytes, the largest amount of ROS is produced in the mitochondria, either through the respiratory chain or diverse mitochondrial proteins like uncoupling proteins (UCPs) or monoamine oxidases (MAOs) [2]. Monoamine oxidases (MAOs) are located at the mitochondrial outer membrane and oxidize amines, resulting in the formation of the ROS molecule hydrogen peroxide (H_2_O_2_). Both isoforms, MAO-A and -B, are expressed in the heart. The isoforms have different substrate specificities. In common, they metabolize neurotransmitters and dopamine, whereas MAO-A uses serotonin and MAO-B uses β-phenylethylamine (PEA) or 1-N-methylhistamine (1N-Met), a histamine metabolite stored in sympathetic nerves, as specific substrates. In heart failure patients, these substrates are increased due to enhanced sympathetic activity, releasing, for example, catecholamines or 1N-Met [3,4,5]. The contribution of MAO-generated ROS to heart disease has been demonstrated in several studies. In spontaneously hypertensive rats (SHRs) [6], or in rats with pulmonary hypertension [7], MAO-inhibitors were able to reduce cardiac hypertrophy. Furthermore, in animal models of myocardial infarction MAO inhibitors reduced infarct size and improved cardiac function [3,8]. The harmful effects of MAO-A were shown by the reduction in the contractile function of cardiomyocytes after the addition of serotonin as an MAO-A substrate [9]. The contribution of MAO-B to cardiac disease processes has also been demonstrated with the help of MAO-B-specific inhibitors or MAO-B knockout mice. Mice lacking MAO-B revealed preserved left ventricular function and reduced cardiac fibrosis when subjected to pressure overload due to transverse aortic constriction (TAC) [10]. In a rat model undergoing myocardial infarction due to permanent ligation of the left anterior descending coronary artery, a MAO-B-selective inhibitor reduced oxidative stress, preserved cardiac function and prevented tissue fibrosis [11]. In addition to studies using global MAO-B knockout mice, reductions in infarct size were demonstrated in mice with inducible and cardiomyocyte-specific MAO-B knockout in vivo [12] and in Langendorff-perfused isolated hearts [13]. Interestingly, in mitochondria, isolated from the whole left ventricle of cardiomyocyte-specific MAO-B-KO mice, ROS production was not completely abolished after the addition of the MAO-B-specific substrate PEA. This suggests, that, in addition to cardiomyocytes, another cell type contributes to MAO-B-dependent ROS production in the heart.

Fibroblasts, which make up about 27% of the cells in the adult heart [14], could play a role in this process. In heart failure or after myocardial infarction, the number of fibroblasts increases. They react strongly to ROS [15] and undergo myofibroblast transdifferentiation, making them the major effector cells in cardiac fibrosis and repair [16,17,18]. However, when excessive expansion of fibrosis occurs, remodeling turns into an adverse process, and ventricular dilation and dysfunction develop [19]. Myofibroblasts are marked by the expression of genes encoding extracellular matrix modulating proteins, like Col1a1, periostin, and fibronectin, as well as alpha-smooth muscle actin (αSMA). Deletion of periostin-expressing myofibroblasts reduces collagen production and scar formation after myocardial infarction [20], thereby indicating the detrimental effects of myofibroblast differentiation. An influence of MAOs on myofibroblast differentiation was already shown by Monroe and Anderson [21]. They demonstrated that norepinephrine influences the expression and secretion of collagens I/III, and other markers of profibrotic activation, via an adrenergic-independent redox pathway in cardiac fibroblasts involving the MAO-mediated generation of ROS. Furthermore, MAO-A and -B were detected in human fibroblasts [22]. However, a direct effect of MAO-B-dependent ROS production in cardiac fibroblasts on myofibroblast differentiation has not been demonstrated yet. We therefore investigated whether the stimulation of MAO-B in cardiac fibroblasts induces ROS production and myofibroblast development, and whether these responses are absent in fibroblast-specific MAO-B knockdown cells.

## 2. Materials and Methods

### 2.1. Generation and Induction of Conditional, Fibroblast-Specific MAO-B KD Mice

The investigation conforms to Guide for the Care and Use of Laboratory Animals published by the US National Institutes of Health (NIH Publication No. 85–23, revised 1996). Animal studies were approved by the Regierungspräsidium Gießen, Germany (registration-no. V 54—19 c 20 15 h 01 GI 20/1 Nr. G 32/2020).

Conditional, fibroblast-specific MAO-B KD mice were generated by crossing mice expressing fibroblast-specific, tamoxifen-inducible Cre recombinase [Tg(Col1a2-cre/ERT,-ALPP)7Cpd/J = Col1a2-CreER^T^] with a conditional MAO-B KO mouse [(B6.129/B6V6.5-MAO-btm1a(KOMP)Wtsi = MAO-B^fl/fl^)] [13] containing MAO-B with exon 5 flanked by loxP sites (Col1a2-CreER^T^x MAO-B^flox/flox^ mouse = FB-MAO-B KD).

Fibroblast-specific deletion of MAO-B was induced by injection (i.p.) of tamoxifen (100 mg/kg body weight) for ten consecutive days followed by a ten-week waiting period. Thereafter, fibroblasts were isolated and cultured. Injections were performed on male and female MAO-B^flox/flox^ mice (referred to as the controls in the following text), as well as on MAO-B knockdown mice, starting at 16–20 weeks of age. The knockdown of MAO-B was monitored by real-time RT-PCR and Western blot analysis.

### 2.2. Isolation of Cardiac Fibroblasts and Cardiomyocytes

Mice were anesthetized by isoflurane inhalation. After cervical dislocation, hearts were extracted and retrograde-perfused in a Langendorff apparatus with a collagenase-containing calcium-free buffer (100 mM NaCl, 2.6 mM KCl, 1.2 mM KH_2_PO_4_, 1.2 mM MgSO_4_·7H_2_O, 25 mM HEPES, and 11 mM glucose; pH 7.4 with NaOH, sterile filtered) at 37 °C. Hearts were cut and cardiomyocytes were pelleted by centrifugation (400 rpm) while fibroblasts remained in the supernatant.

The cell pellet was resuspended in medium and readjusted to physiological calcium concentrations. Cardiomyocytes were then plated on laminin-coated dishes in culture medium (118 mM NaCl, 1.2 mM KH_2_PO_4_, 4.7 mM KCl, 5 mM glucose, 0.8 mM MgSO_4_, 2.5 mM CaCl_2_-dihydrat, 1.9 mM Na-pyruvat, and 10 mM Hepes, at pH 7.4) and cultured at 37 °C in a carbon dioxide (5.5%) incubator for 45 min before harvest.

The fibroblast containing supernatant was centrifugated at 1500 rpm for five minutes. Pelleted cells were suspended in fibroblast growth medium (PromoCell, Heidelberg, Germany), containing 2% penicillin/streptavidin, plated on 10 cm culture dishes, and incubated in a carbon dioxide (5%) incubator at 37 °C. After selective attachment of fibroblasts within 1 h, dishes were washed with and cultured in fibroblast growth medium, including 2% penicillin/streptavidin and 10% FCS. Until confluence was reached, the medium was changed every three days. All fibroblasts were used in the first passage (P1).

### 2.3. Real-Time RT-PCR

Total RNA was isolated using Trizol (Invitrogen, Waltham, MA, USA). After RNA isolation DNAse treatment was performed. Then, RNA was reverse transcribed with a QuantiTect Reverse Transcription Kit from Qiagen (Hilden, Germany). Real-time RT-PCR was performed using SYBR Green fluorescence of the Biorad detection system (Bio-Rad, Hercules, CA, USA). For each primer pair the linear amplification range was verified. RNA expression was determined according to the 2^−ΔΔCt^ method, as described in [23]. Bestkeeper, which calculated a correlation of the housekeeping genes (18SrRNA, B2M, hypoxanthine-guanine phosphoribosyltransferase (HPRT), glyceraldehyde 3-phosphate dehydrogenase (GAPDH), and actin), was used as the internal housekeeping reference [24]. The primer sequences are listed in Table 1.

### 2.4. Western Blot

Cells were lysed in RIPA buffer and subsequently centrifuged at 13,000× *g* for 10 min. The protein concentration of the supernatant was determined using the DC Protein Assay kit (BioRad, Hercules, CA, USA). Equal amounts of total proteins were electrophoretically separated on NuPage 10% Bis-Tris Gel (Thermo Scientific, Rockford, IL, USA) and transferred to nitrocellulose membranes. For the detection of various proteins on one membrane, the membranes were cut between protein bands of interest. Western blots were performed using a standard protocol, with specific primary antibodies against MAO-B (#M1821, SIGMA, Roedermark, Germany), MAO-A (#ab126751, Abcam, Cambridge, UK), Col1a1 (#PA5-95137, Invitrogen, Thermo Scientific, Rockford, IL, USA), and GAPDH (#5G4Mab6C5, Hy Test Ltd., Turku, Finland). Anti-mouse IgG or anti-rabbit IgG, both HRP-linked antibodies, were used as the secondary antibodies (Cell Signaling Technology, Frankfurt am Main, Germany). Immuno-reactive bands were detected using the SuperSignal West Femto Maximum Sensitivity Substrate or SuperSignal West Pico Chemiluminescent Substrate (Thermo Scientific, Rockford, IL, USA). Protein bands were quantified with Quantity One software Version 4.6.9 (Bio-Rad Laboratories, Raleigh, NC, USA) or ImageJ Version 1.53m. GAPDH was used for normalization.

### 2.5. Immunohistology

In total, 5000 fibroblasts (P1) were plated on coverslips and cultured for 2 days. Then, cells were fixed with 4% paraformaldehyde. The blocking solutions were 10% BSA, 0.001% Tween in PBS, and mouse IgG blocking solution (Thermo Fisher, Rockford, IL, USA). The primary antibody DDR2 (Santa Cruz, sc-81707, 1:100, Heidelberg, Germany) was used, followed by the secondary antibody goat anti-mouse TRITC (Invitrogen A-11003, 1:100, Thermo Fisher, Rockford, IL, USA). In addition, nuclei were stained with 4′,6-Diamidino-2-phenylindol-dihydrochlorid (DAPI, 100 ng/mL PBS). Finally, slides were semi-permanently sealed with Fluoromount (Sigma, Merck, Darmstadt, Germany). Pictures of the cells were taken with the fluorescence microscope BZ-X800 (Keyence, Frankfurt am Main, Germany).

### 2.6. Measurement of ROS in Isolated Cardiac Mitochondria

Mitochondria were isolated from the whole left ventricle. Therefore, the ventricle was minced in isolation buffer containing sucrose (250 mM), HEPES (10 mM), and EGTA (1 mM) and homogenized with a 15 mL glass Potter, as described before [13]. A total of 25 µg of freshly isolated mitochondria was used for the detection of ROS hydrogen peroxide by Amplex Ultra Red staining (A36006; Invitrogen, Waltham, MA, USA). Fluorescence was measured continuously for ten minutes with excitation and emission wavelengths of 565 and 581 nm, respectively, in a Care Eclipse spectrophotometer (Agilent, Santa Clara, CA, USA). Glutamate (5 mM) and malate (2.5 mM) were used as substrates for complex I, as described before [13].

### 2.7. Detection of Mitochondrial ROS Production in Fibroblasts

In total, 10,000 fibroblasts (P1) per well were plated on six-well plates and cultured until confluence in fibroblast growth medium, including 2% penicillin/streptavidin and 10% FCS. Fibroblasts were prestained with 1 µM MitoSOX™ Red (Invitrogen, Thermo Fisher, Rockford, IL, USA), a fluorogenic dye for the highly selective detection of superoxide in the mitochondria of live cells. Subsequently, cells were stimulated with PEA for 10 min. Imaging was done with the BZ-X800 fluorescence microscope (Keyence, Frankfurt am Main, Germany) using the MitoSox filter cube (excitation and emission wavelengths of 396 nm and 610 nm, respectively) (Chroma, Bellows Falls, VT, USA). The increased brightness of the cells, due to enhanced red fluorescence caused by mitochondrial ROS, was evaluated using the BZ-X800 analyzer software, version 1.1.1.8.

### 2.8. Migration Assay

In total, 10,000 fibroblasts (P1) per well were plated on six-well plates and cultured until confluence in fibroblast growth medium, including 2% penicillin/streptavidin and 10% FCS. A scratch was made through the confluent cell layer using a yellow pipette tip. Then, fibroblasts were stimulated with PEA and microscopic images of the scratched area were immediately taken using the BZ-X800 microscope (Keyence, Frankfurt am Main, Germany, magnification 20×), and 24 and 48 h after PEA stimulation. Migration of cells into the scratch was evaluated using the BZ-X800 analyzer software and expressed as the percentage of fibroblasts that migrated into the scratch in relation to the total scratch area.

### 2.9. Statistics

Data are presented as mean ± SD. Normally distributed data were analyzed with an unpaired 2-tailed Student’s *t*-test (2 groups) or ANOVA, followed by Duncan post hoc test (≥3 groups). SPSS (version 29; SAS Institute Inc., Cary, NC, USA) was used for those calculations. A *p* value <0.05 was considered statistically significant.

## 3. Results

### 3.1. MAO-B KD in Cardiac Fibroblasts

**Figure 1:**
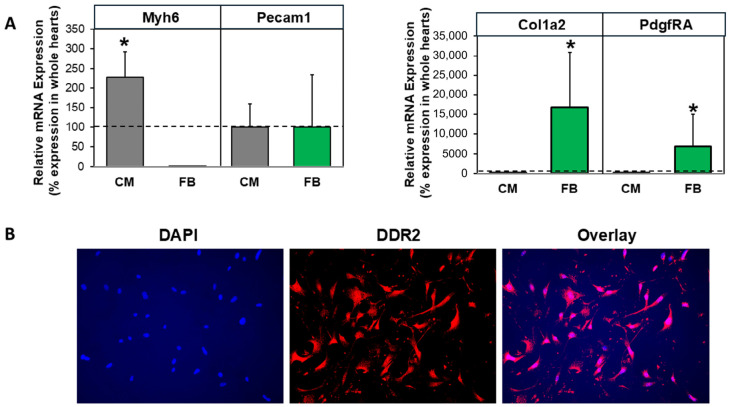
**Characterization of isolated cells from collagenase-digested mouse hearts.** After isolation and cultivation of cardiomyocytes (CMs) and fibroblasts (FBs) from control mice, purity of cultures was verified in (**A**) real-time RT-PCR using specific primers for Myh6 as a cardiomyocyte marker, Pecam1 as an endothelial marker, and Col1a2 and PdgfRA as fibroblast markers. Normalization of relative expression was done with bestkeeper using B2M, HPRT, GAPDH and 18SrRNA as housekeeping genes and expressed as percent of their expression in whole heart tissues (indicated by the dashed line). (n = 6 for whole hearts and n = 11–15 for CMs or FBs, * *p* ≤ 0.05 vs. whole heart expression.) (**B**) Immunofluorescence of fibroblast cultures (magnification 20×) was performed using antibodies against the fibroblast-specific marker protein DDR2 and TRITC-labeled secondary antibodies. DAPI was used for nuclear staining.

Fibroblasts and cardiomyocytes were isolated by collagenase perfusion of mice hearts. The purity of fibroblast cultures was verified in real-time RT-PCR based on cell-type-specific gene expression patterns. As depicted in Figure 1A, the cardiomyocyte-specific marker Myh6 was detected only in cardiomyocyte cultures. In contrast, mRNA expression of the fibroblast marker genes, PdgfRA and Col1a2, was strongly enriched in fibroblast cultures. The expression of Pecam1-mRNA, as an endothelial marker, was neither enriched in cardiomyocytes nor in fibroblast cultures. For the detection of fibroblasts in the cell culture, fibroblasts were plated on coverslips and stained immunohistochemically with DDR2 antibodies. Nuclei were DAPI-stained. As depicted in Figure 1B, all cells were positive for the fibroblast-specific marker DDR2. This shows that fibroblast cultures were pure and without contamination from any other heart cell types. The purity of the fibroblast cultures was a basic prerequisite for our subsequent analyses on fibroblast-specific MAO-B effects.

**Figure 2:**
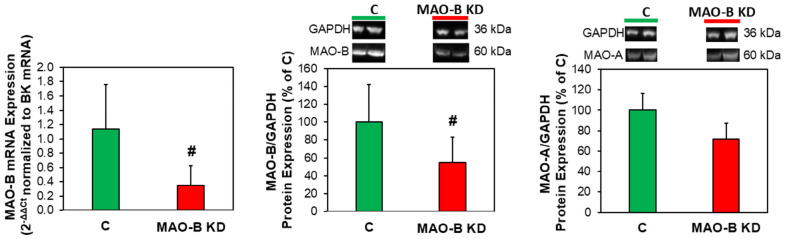
**Reduction in MAO-B expression in fibroblasts after MAO-B knockdown.** After induction of MAO-B KD via tamoxifen injections, fibroblasts were isolated. MAO-B expression was analyzed in real-time RT-PCR (7–11, ^#^ *p* ≤ 0.05 vs. C). MAO-B (n = 9–12, ^#^ *p* ≤ 0.05 vs. C) and MAO-A (n = 5–6) were analyzed in Western blots.

For the induction of MAO-B knockdown, MAO-B KD and control mice were injected with tamoxifen for ten consecutive days, followed by a ten-week waiting period. Then, cardiac fibroblasts were isolated, cultured and harvested in the first passage. MAO-B mRNA expression was reduced to 0.35 ± 0.27-fold in fibroblasts from MAO-B KD mice compared to controls (n = 7–11, *p* ≤ 0.05). On the protein level, a reduction in MAO-B to 55 ± 28% compared to controls was observed (Figure 2). To rule out that knockdown of MAO-B may induce a compensatory upregulation of MAO-A, the protein expression of MAO-A was tested. However, MAO-A expression was not enhanced in MAO-B KD fibroblasts (Figure 2).

### 3.2. ROS Production Is Reduced in Cardiac Mitochondria and Fibroblasts from MAO-B KD Mice

**Figure 3:**
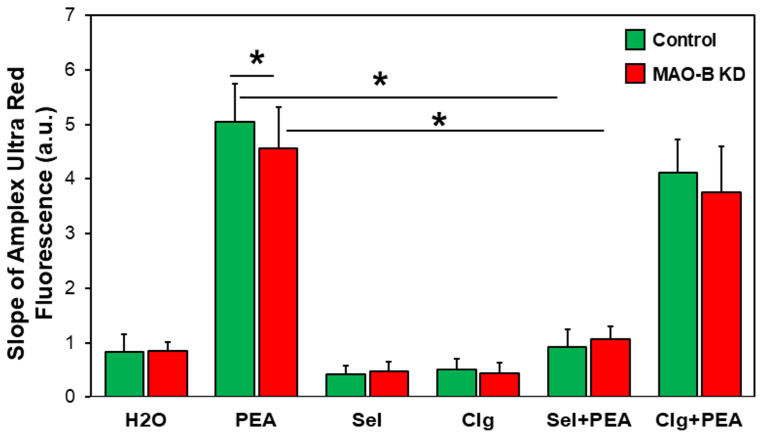
**ROS production of isolated cardiac mitochondria of MAO-B knockdown mice.** Mitochondria of control and MAO-B KD mice hearts were isolated and the ROS production was measured in the presence of complex I substrate and after the addition of PEA (250 µM) and the inhibitor selegiline (Sel, 1 µM) or clorgyline (Clg, 1 µM) (C, n = 7; KD, n = 9, * *p* ≤ 0.05).

To analyze whether the deletion of MAO-B reduced ROS production, mitochondria were isolated from left ventricles (LVs) and ROS production was measured using Amplex-Red. Mitochondria isolated from MAO-B KD and control hearts showed similar ROS production under basal conditions when respiring on complex-I substrate. The addition of the MAO-B substrate PEA increased ROS production. However, the increase in ROS production was significantly lower in the mitochondria of FB-MAO-B KD mice vs. WT (Figure 3). ROS formation could be down-regulated by the MAO-B-specific inhibitor selegiline. The MAO-A-specific inhibitor clorgyline had no effect on ROS production.

**Figure 4:**
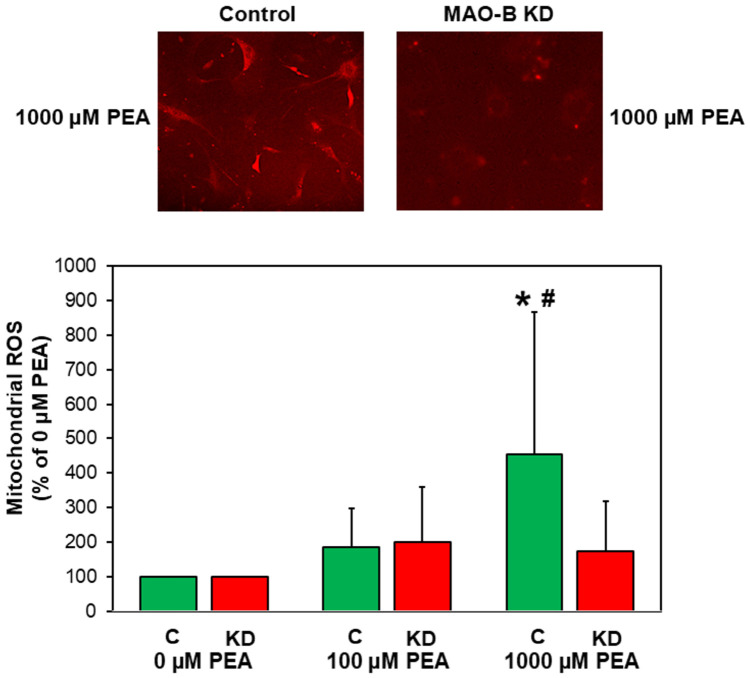
**Reduced mitochondrial ROS production in MAO-B KD fibroblasts.** Fibroblasts were incubated with 1 µM MitoSOX™ Red, followed by PEA stimulation for 10 min. Then, fluorescence images were taken and quantified. (n = 11–16, * *p* ≤ 0.05 vs. unstimulated control, ^#^ *p* ≤ 0.05 vs. KD, 1000 µM PEA.).

To determine, if fibroblasts of MAO-B KD show diminished responses upon stimulation with MAO-B-specific substrates, cells were stimulated with two different concentrations of β-phenylethylamine (100 and 1000 µM PEA). Under these conditions, mitochondrial ROS production was measured using MitoSOX™ Red. Ten minutes after the addition of 1000 µM PEA, red fluorescence, indicating mitochondrial ROS production, increased to 455 ± 413% in controls vs. unstimulated cells (n = 12, *p* ≤ 0.05), whereas fibroblasts isolated from MAO-B KD mice did not show a significant increase in red fluorescence under stimulation with 1000 µM PEA compared to unstimulated control levels (Figure 4). Compared to PEA-stimulated controls, the response of MAO-B KD fibroblasts to stimulation with 1000 µM PEA was significantly reduced to 173 ± 144% (n = 12–15, *p* ≤ 0.05).

### 3.3. Reduced Myofibroblast Differentiation in MAO-B KD Fibroblasts

**Figure 5:**
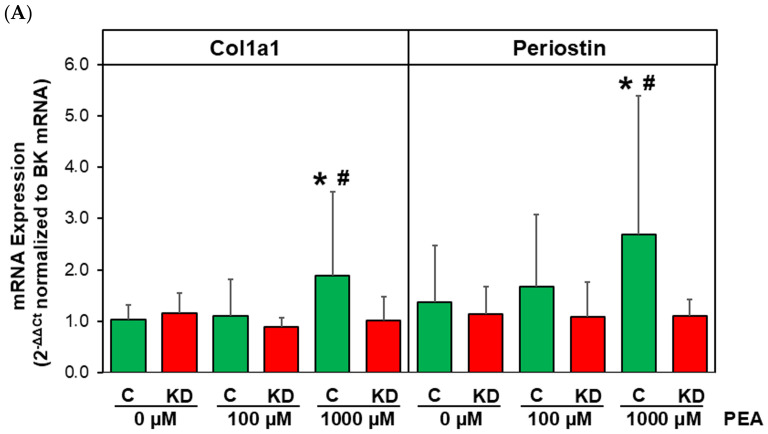

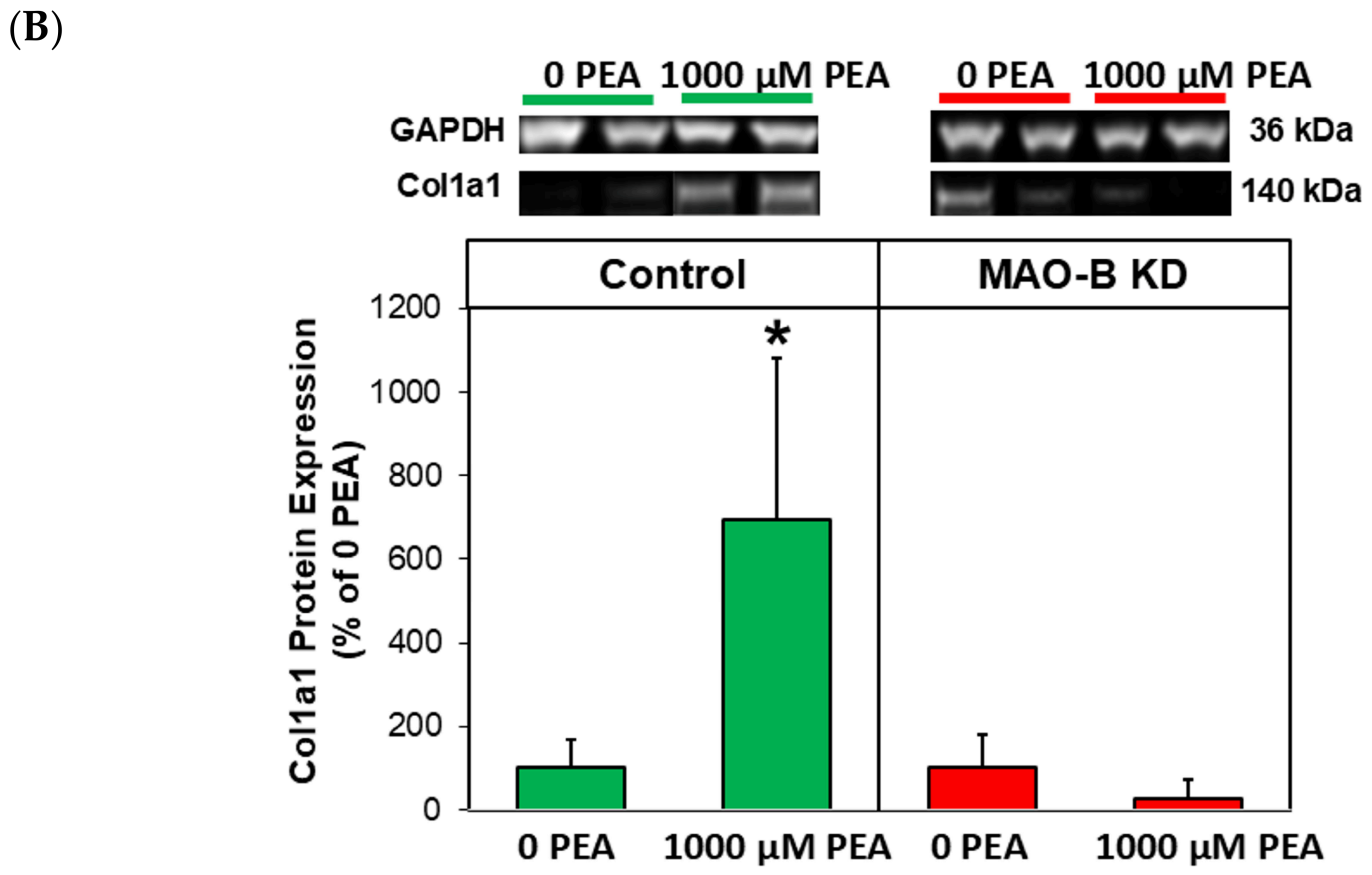
**Myofibroblast marker genes are not increased under PEA stimulation in MAO-B KD fibroblasts.** Fibroblasts were stimulated with PEA. (**A**) After 24 h, RNA was isolated and real-time RT-PCR was performed for detection of Col1a1 and periostin mRNA expression. Normalization of relative expression was done with bestkeeper using actin, HPRT, GAPDH and 18SrRNA as housekeeping genes. (n = 7–12, * *p* ≤ 0.05 vs. unstimulated control, ^#^ *p* ≤ 0.05 vs. KD, 1000 µM PEA.) (**B**) After 48 h, fibroblasts were isolated and Col1a1 protein expression was analyzed in Western blots. GAPDH was used as a loading control (n = 6, *p* ≤ 0.05 vs. 0 PEA).

ROS production in vivo is a well-known trigger of myofibroblast differentiation. Therefore, we tested if PEA stimulation initiates myofibroblast differentiation in isolated fibroblasts. As depicted in Figure 5A, mRNA expression of the myofibroblast marker genes Col1a1 and periostin increased under the stimulation of controls with 1000 µM PEA for 24 h. This response in myofibroblast marker gene expression was absent in fibroblasts of MAO-B KD mice. Similarly, Col1a1 protein expression increased to nearly 700% compared to unstimulated controls. This response was absent in MAO-B KD fibroblasts after PEA stimulation (Figure 5B).

**Figure 6:**
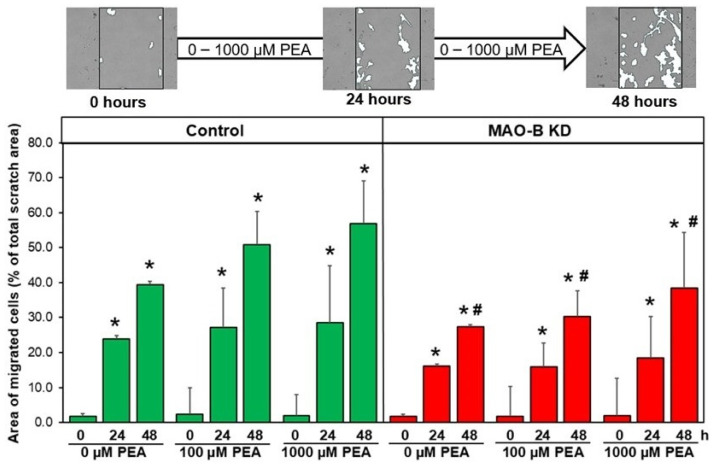
**Reduced migration potential of MAO-B KD fibroblasts.** In a confluent monolayer of fibroblasts, a scratch was made and first pictures were taken (0 h). Then, fibroblasts were stimulated with PEA (100 and 1000 µM). Pictures were taken at the same location before and 24 and 48 h after stimulation. The area of migrating cells into the scratch area was related to the initial scratch area. (n = 6–9, * *p* ≤ 0.05 vs. time point 0 h, ^#^ *p* ≤ 0.05 vs. related 48 h control value).

Another criterion for myofibroblast differentiation is the ability of fibroblasts to migrate. In order to test fibroblast motility, we assessed the movement of fibroblasts into a scratch area created in a confluent cell layer. As depicted in Figure 6, under stimulation with PEA, a dose-dependent increase in fibroblast motility was seen in controls and MAO-B KD fibroblasts. However, the response to PEA of fibroblasts from MAO-B KD mice was significantly reduced compared to time- and concentration-matched controls. Under stimulation with 1000 µM PEA, 57 ± 12% of the scratch was covered with fibroblasts that migrated into the scratch area in controls after 48 h, whereas only 38 ± 16% of the scratch area was covered with migrated MAO-B-KD fibroblasts (n = 6–9, *p* ≤ 0.05). Interestingly, neither enhancement of proliferation nor cell death was observed in fibroblasts under PEA stimulation, since the number of fibroblasts, counted by nuclear staining with DAPI, did not change under stimulation with 1000 µM PEA in controls (91 ± 38% vs. unstimulated cells, n = 6, n.s.) or in MAO-B KD (99 ± 15% vs. unstimulated cells, n = 6, n.s.).

## 4. Discussion

The main findings of this study are that the stimulation of MAO-B in cardiac fibroblasts enhanced mitochondrial ROS production and increased myofibroblast differentiation. This indicates that, in cardiac disease, not only cardiomyocytes but also fibroblasts can contribute to MAO-B-dependent ROS formation and cardiac damage.

The contribution of ROS to cardiac damage is a well-known phenomenon. MAOs have been described as central enzymes involved in cardiac ROS production [2]. Due to the use of pharmacologic inhibition or general KO techniques of MAOs, the cellular phenotype producing ROS in the heart was not identified clearly. Just recently, our working group created a cardiomyocyte-specific MAO-B knockout [13]. Reductions in infarct size were observed in vivo [12], as well as in Langendorff-perfused hearts of these animals [13], thereby demonstrating that MAO-B is activated under ischemic conditions, and contributes to ischemia/reperfusion injury via mitochondrial ROS production in cardiomyocytes. However, despite MAO-B KO in cardiomyocytes, in mitochondria, isolated from the whole left ventricle of these mice, ROS production was not completely abolished after the addition of the MAO-B-specific substrate PEA. ROS production remained significantly, albeit only slightly, elevated following PEA treatment of the mitochondria from these knockout hearts [13]. This remaining small increase in ROS formation in mitochondria from cardiomyocyte-specific MAO-B KO hearts was most likely caused by mitochondria from non-cardiomyocytes and correlates well with the now presented small but significant decrease in ROS production (Figure 3) when using mitochondria isolated from whole ventricles of fibroblast-specific MAO-B knockdown mice. Since cardiomyocytes have the largest cellular portion and are the most mitochondria-rich cells in the heart, the main part of mitochondria isolated from ventricles should be of cardiomyocyte origin, which is not affected by the MAO-B knockdown in fibroblasts. This explains the small effect of PEA on ROS production in mitochondria from ventricles of fibroblast-specific MAO-B KD mice. All other experiments of the study were done in isolated cardiac fibroblasts. The treatment of the animals with tamoxifen resulted in the reduction in MAO-B expression in isolated fibroblasts by around 50% at the mRNA and protein levels. Measurement of ROS in pure cardiac fibroblast cultures (Figure 4) revealed a strong decline in ROS production upon MAO-B knockdown. This substantiates the role of MAO-B in the formation of ROS in cardiac fibroblasts.

For the analysis of MAO-B-specific effects, we incubated fibroblasts with PEA, which is specifically metabolized by MAO-B, but not MAO-A. PEA is a trace amine that occurs naturally in the body. Physiological concentrations in the nanomolar range are described in [25]. However, in experiments with isolated tissues or cells, responses to PEA only occur at concentrations multiple orders of magnitude above normal PEA levels [25]. Neuromodulatory actions were produced at submicromolar levels, whereas cardiovascular or kidney responses emerge at micromolar concentrations [26]. In isolated arteries of humans, for example, PEA provokes contractions when administered cumulatively in concentrations from 1 to 3000 µM [27], which is equivalent to the PEA levels used in our experiments on fibroblasts. Furthermore, the specificity of PEA in µmolar ranges is evident in Figure 2, since ROS production in isolated mitochondria was abrogated by the MAO-B-selective inhibitor selegiline, but not by MAO-A-specific clorgyline. Further evidence for specific responses in myofibroblast differentiation comes from the absence of enhanced collagen1a1 expression or reduction in migration potential upon PEA treatment of MAO-B KD fibroblasts.

MAO-B inhibition has been described in several in vivo animal studies as a cardioprotective intervention in myocardial infarction [11,12,28]. Improved cardiac function under MAO-B inhibition was observed in these studies, along with reductions in radical stress, cardiomyocyte hypertrophy and apoptosis, and interstitial fibrosis. While in cardiomyocytes, the induction of cell death via MAO-B was observed [29], we now demonstrate that MAO-B in cardiac fibroblasts initiates their transition into myofibroblasts as the main mechanism of MAO-B action in this cell type. This process is marked by the expression of genes encoding extracellular matrix-modulating proteins, like Col1a1 and periostin. Myocardial fibrosis is considered as a main cause for post-myocardial infarction ventricular remodeling, resulting in ventricular stiffness and dysfunction. Interestingly, the in vivo MAO-B-dependent induction of Col1a1 has also been shown after myocardial infarction [11]. The deletion of periostin-expressing myofibroblasts reduced collagen production and scar formation after myocardial infarction [20], thereby showing the detrimental effects of myofibroblast differentiation on heart function. Thus, our findings of MAO-B-dependent myofibroblast formation reveal evidence for the contribution of this process to cardiac damage.

There is a very close interaction between cardiomyocytes and fibroblasts in vivo, since fibroblasts express connexins and are connected to cardiomyocytes via gap junctions [30], enabling electrical coupling [31]. Therefore, besides the action of MAO-B in fibroblasts in vitro, as shown in this study, we cannot rule out that the ROS production after myocardial infarction in fibroblasts amplifies detrimental effects on cardiomyocytes or vice versa. Thus, fibroblast–cardiomyocyte crosstalk may influence cardiac disease development, and MAO-B in fibroblasts does modulate the fibrotic remodeling process but may also impact cardiomyocyte function. This should be analyzed in future studies.

### Limitations of the Study

The limitations of our study are that we did not achieve a complete MAO-B knockout due to tamoxifen treatment, and that the MAO-B-specific substrate PEA was used in the micromolar range, whereas physiologic PEA concentrations measured at steady state are in the nanomolar range. Although we were able to demonstrate that responses in ROS production and myofibroblast differentiation are reduced in MAO-B KD fibroblasts and that MAO-B KD does not lead to a compensatory increase in MAO-A expression, we cannot rule out the possibility that MAO-A also contributes to myofibroblast differentiation. However, we did not use an MAO-A-specific substrate and thus focused exclusively on MAO-B-specific effects. Furthermore, our findings regarding the contribution of MAO-B to myofibroblast differentiation at the cellular level must be confirmed in vivo using a cardiac remodeling model.

## 5. Conclusions

In conclusion, using fibroblast-specific MAO-B KD mice, we demonstrate that MAO-B in cardiac fibroblasts can be activated by specific substrates, thereby stimulating mitochondrial ROS and myofibroblast differentiation. These processes can provoke massive cardiac fibrosis, i.e., after myocardial infarction in vivo. Therefore, MAO-B activation should be prevented in cardiomyocytes and fibroblasts to protect the heart from damage.

## Figures and Tables

**Table 1:** PCR primers.

18SrRNA	5′-TGCGGCGGCGTTATTCCCAT-3′ 5′-TGGTGGTGCCCTTCCGTCAA-3′
Actin	5′-CCTGAACCCTAAGGCCAACCGT-3′ 5′-TGTAGCCACGCTCGGTCAGGAT-3′
B2M	5′-GCTATCCAGAAAACCCCTCAA-3′ 5′-CATGTCTCGATCCCAGTAGACGGT-3′
Col1a1	5′-AGCACGTCTGGTTTGGAGAG-3′ 5′-GACATTAGGCGCAGGAAGGT-3′
Col1a2	5′-CTAGCCAACCGTGCTTCTCA-3′ 5′-CAACATCGTTGGAACCCTGC-3′
GAPDH	5′-ACGGCACAGTCAAGGCCGAG-3′ 5′-CACCCTTCAAGTGGGCCCCG-3′
Hprt	5′-ATGGACAGGACTGAAAGACTTG-3′ 5′-AATCCAGCAGGTCAGCAAAG-3′
MAO-B	5′-AGATGGGCCAAGAGATTCCC-3′ 5′-CCCTGTCTGGTCAATGTGGA-3′
Myh6	5′-GGATGACGTCACCTCCAACA-3′ 5′-AGCTTCACGCGGTACTCATT-3′
Periostin	5′-GCAAACCACTTTCACCGACC-3′ 5′-CGTTGGTCCATGCTCAGAGT-3′
PdgfRA	5′-GAGATCGCTGTACGATCGGC-3′ 5′-AAGTCAGACCCTCGGAGTCA-3′
Pecam 1	5′-CACACCGAGAGCTACGTCAT-3′ 5′-TTGGATACGCCATGCACCTT-3′

## Data Availability

We will publish the original data presented in the study on FigShare once the manuscript is published. The original data presented in the study are openly available in FigShare, DOI 10.6084/m9.figshare.31899133.

## Abbreviations

The following abbreviations are used in this manuscript:

CM	Cardiomyocyte
Col1a1	Collagen type 1
DAPI	4′,6-Diamidino-2-phenylindol-dihydrochlorid
DDR2	Discoidin domain receptor 2
FB	Fibroblast
KD	Knockdown
KO	Knockout
MAO-B	Monoamine oxidase B
Myh6	Myosin Heavy Chain 6
PDGFRA	Platelet-derived growth factor receptor alpha
PEA	β-phenylethylamine
Pecam1	Platelet endothelial cell adhesion molecule 1
ROS	Reactive oxygen species
WT	Wild type
